# Disulfidptosis vs. Ferroptosis: A Comprehensive Review of SLC7A11-Mediated Metal Dyshomeostasis and Cell Death

**DOI:** 10.3390/biom16050671

**Published:** 2026-05-01

**Authors:** Iogann Tolbatov, Alessandro Marrone

**Affiliations:** 1 Department of Chemical, Physical, Mathematical and Natural Sciences, University of Sassari, 07100 Sassari, Italy; 2 Department of Pharmacy, University “G. D’Annunzio” of Chieti-Pescara, Via dei Vestini 31, 66100 Chieti, Italy; amarrone@unich.it

**Keywords:** disulfidptosis, ferroptosis, SLC7A11 (xCT), metal dyshomeostasis, cuproptosis, cancer metabolism

## Abstract

This systematic review examines the emerging interplay between ferroptosis and disulfidptosis, two distinct forms of regulated cell death (RCD) centered on the SLC7A11 (also known as xCT)-mediated metabolic paradox. Traditionally recognized as a potent anti-ferroptotic factor, SLC7A11 imports cystine for glutathione synthesis to neutralize iron-dependent lipid peroxidation. However, the discovery of disulfidptosis identifies SLC7A11 as a metabolic liability, representing a paradigm shift in our understanding of cellular antioxidant defense. This discovery reveals a transformative vulnerability in SLC7A11-overexpressing cells, shifting the focus from conventional survival mechanisms to the consequences of catastrophic structural collapse. Beyond metabolic exhaustion, this review highlights the role of metal dyshomeostasis as a primary driver, spanning from iron-catalyzed ferroptosis to copper-mediated metabolic interference. This conceptual framework redefines the SLC7A11 axis as a targetable “double-edged sword” in therapy-resistant malignancies. Clinical synthesis of multi-omic gene signatures, such as the disulfidptosis- and ferroptosis-related gene prognostic score (DRGPS) and the ferroptosis- and disulfidptosis-related gene (FDRG) scores, demonstrates their robust value in prognostic stratification and in predicting immunotherapy response across malignancies, including lung adenocarcinoma and hepatocellular carcinoma. Furthermore, we evaluate the capacity of disulfidptosis to prime immunogenic cell death (ICD) and remodel the immunosuppressive tumor microenvironment to bypass chemoresistance. By integrating mechanistic insights with clinical data, this review provides a comprehensive framework for targeting the SLC7A11 axis as a transformative therapeutic vulnerability in precision oncology.

## 1. Introduction

The landscape of regulated cell death (RCD) has expanded significantly with the characterization of ferroptosis, an iron-dependent process defined by the lethal accumulation of lipid peroxides [1,2,3]. This form of cell death is primarily driven by the convergence of iron overload and the collapse of cellular antioxidant defenses, specifically the solute carrier family 7 member 11 (SLC7A11, also known as xCT)-glutathione (GSH)-glutathione peroxidase 4 (GPX4) axis [4,5,6,7]. Conversely, the recent identification of disulfidptosis has introduced a distinct RCD regulatory program triggered by acute disulfide stress [8,9,10]. Unlike ferroptosis, disulfidptosis occurs specifically in cells harboring high levels of SLC7A11 under metabolic constraints [8,9,11,12]. Notably, this death mode is biochemically unique; it remains unaffected by standard inhibitors of apoptosis, ferroptosis, or necroptosis, yet it is specifically reversible through the administration of disulfide-reducing agents such as β-mercaptoethanol (2-ME) or tris(2-carboxyethyl)phosphine (TCEP) [13,14,15] (Table 1).

**Figure 1:**
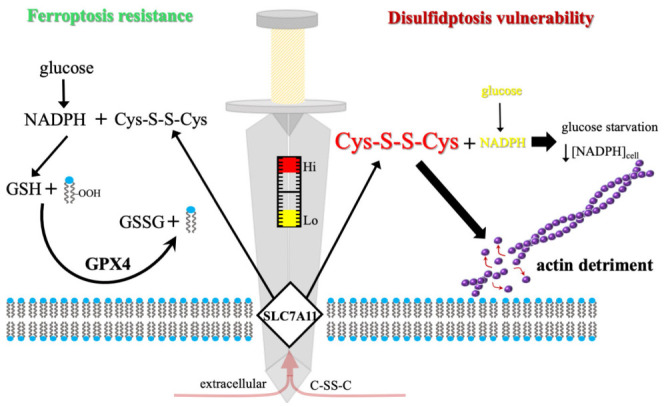
The SLC7A11 metabolic pivot: a sword-edge between survival and disulfidptosis. The central sword represents the metabolic scale upon which cellular fate is balanced, with the cystine transporter SLC7A11 serving as the membrane-bound pivot. The concentration of reducing equivalents (NADPH) determines whether the system tips toward homeostasis or catastrophic collapse. **Left (homeostasis/glucose sufficiency):** under normal metabolic conditions, high levels of glucose feed the PPP to ensure a robust supply of NADPH. This reductive power allows the SLC7A11-imported cystine (C-SS-C) to be efficiently converted into cysteine and subsequently GSH. GPX4 utilizes this GSH to maintain redox balance and prevent membrane damage, ensuring cell survival. **Right (disulfidptosis/glucose starvation):** upon glucose starvation (indicated by the metabolic explosion), the source of reductive power is severed, leading to NADPH depletion. Without NADPH, the imported cystine cannot be reduced, leading to a toxic C-SS-C excess. This unreduced cystine induces acute disulfide stress, which targets the F-actin cytoskeleton. The resulting aberrant cross-linking triggers actin detriment, leading to structural contraction and the execution of disulfidptosis.

The discovery of disulfidptosis has fundamentally reconfigured our understanding of SLC7A11, which was traditionally characterized almost exclusively as a potent suppressor of ferroptosis [16,17,18]. By mediating the exchange of extracellular cystine for intracellular glutamate, SLC7A11 provides the rate-limiting precursor, cysteine, for GSH synthesis. This enables GPX4 to neutralize lipid hydroperoxides and maintain membrane integrity [19,20,21]. However, emerging evidence suggests that the very transport capacity required for antioxidant defense becomes a liability under metabolic pressure, transforming SLC7A11 into a “double-edged sword” [8,9] (Figure 1). While robust expression confers resistance to ferroptosis, it simultaneously imposes a profound metabolic dependency that can be strategically exploited for therapeutic gain [22]. As established in the following sections, this transition from a survival factor to a lethal driver is governed by the cell’s underlying reductive capacity and its ability to manage a systemic disulfide burden [23,24,25].

## 2. Mechanistic Comparison—Triggers, Metabolism, and Morphology

The divergence between ferroptosis and disulfidptosis is governed by distinct metabolic constraints and the resulting physical manifestations of cellular damage (Table 2). While ferroptosis is fundamentally an iron-dependent process triggered by GSH depletion, GPX4 inactivation, or excessive iron accumulation [20,26,27], its execution relies on iron-mediated Fenton reactions that generate ROS to catalyze lethal lipid peroxidation [28,29,30,31,32]. In sharp contrast, the primary driver of disulfidptosis is acute disulfide stress rather than oxidative lipid damage. As expanded upon in Section 3, this modality originates from a systemic failure of the cellular reductive machinery to process imported cystine [8,9,33,34]. This marks a metabolic “point of no return” characterized by the catastrophic accumulation of intracellular disulfides, a process distinct from the iron-mediated lipid peroxidation of ferroptosis.

This distinction is most visible in the contrasting dynamics of NADPH and GSH. Disulfidptosis is critically defined by an acute NADPH deficit [8,31,37]. While NADPH is also utilized in ferroptosis to regenerate GSH, its rapid and total exhaustion is the definitive hallmark of disulfidptosis [8,29]. Furthermore, although GSH levels often decline during disulfidptosis, the cell death is not a direct result of GSH depletion. Instead, it stems from disulfides forming aberrant intermolecular bonds between structural proteins [9,10,38]. This creates a notable “cystine paradox”: while cystine starvation promotes ferroptosis by cutting off the GSH supply, it actually suppresses disulfidptosis by preventing the accumulation of the very disulfides that execute the pathway [8,9] (Table 2).

**Figure 2:**
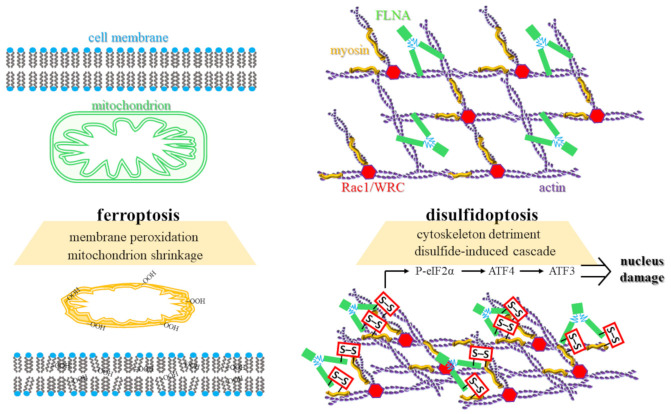
Comparative topographies of ferroptotic and disulfidptotic execution: organelle-level oxidation versus cytoskeletal collapse. The figure delineates the divergent “points of no return” for two distinct RCD programs, contrasting the mitochondrial and membrane failure of ferroptosis with the systemic structural detriment of disulfidptosis. **Left (Ferroptosis):** The execution of ferroptosis is centered on localized organelle-level damage and lipid peroxidation. The transition from homeostasis to death is characterized by the transformation of healthy mitochondria, possessing expansive cristae and a clear intermembrane space, into a shrunken mitochondrion state. This morphological hallmark involves increased mitochondrial matrix density and the total loss of internal cristae. The process is catalyzed by iron-mediated ROS generation, leading to the accumulation of lipid hydroperoxides (-OOH) within the plasma membrane. This oxidative attack disrupts the highly organized polyunsaturated fatty acid (PUFA) bilayer, resulting in membrane permeabilization and the physical rupture of the cell. **Right (Disulfidptosis):** The execution of disulfidptosis is defined by the catastrophic remodeling of the structural framework. Under physiological conditions, the F-actin cytoskeleton is organized into a branched network scaffolded by the Rac1/WRC complex and FLNA. Upon the induction of acute disulfide stress, the exhaustion of cellular NADPH prevents the reduction of imported cystine, leading to the aberrant formation of intermolecular disulfide bonds (S-S) between structural proteins such as actin, myosin, and FLNA. This induces a jagged contraction of the F-actin filaments and systemic cytoskeletal collapse. This structural failure triggers a retrograde stress signal to the nucleus via the P-eIF2α → ATF4 → ATF3 axis, visually represented by the signaling cascade and nuclear detonation, marking the irreversible execution of the disulfidptotic program.

The physical signatures of these death modes are equally divergent (Figure 2). Ferroptotic cells typically exhibit shrunken mitochondria with increased membrane density, a reduction or disappearance of cristae, and ruptured outer mitochondrial membranes [39,40,41]. Conversely, disulfidptosis is characterized by dramatic remodeling of the actin cytoskeleton [8,9]. The accumulation of disulfide bonds in structural proteins, such as filamin A (FLNA) and non-muscle myosin II (MYH9), precipitates F-actin network contraction, systemic cytoskeletal collapse, and the detachment of the actin filaments from the plasma membrane [8,9,42,43]. As detailed in Section 3, this process occurs independently of cellular energy depletion or physical crystallization [8,9,44].

Furthermore, these pathways intersect with broader cellular stress responses and regulatory complexes. Disulfidptosis is intimately linked to endoplasmic reticulum (ER) stress, stemming from the organelle’s role as the primary hub for oxidative protein folding. Mechanistically, the exhaustion of the NADPH pool and the resulting accumulation of unreduced cystine within the ER lumen deplete the local reductive capacity required to resolve non-native disulfide bonds. This disruption prevents the proper folding of nascent polypeptides, leading to a systemic impairment of proteostasis. This failure activates the phosphorylated eukaryotic initiation factor 2 alpha (P-eIF2α)/activating transcription factor 4 (ATF4)/activating transcription factor 3 (ATF3) signaling axis [35,45]. Within the causal hierarchy of disulfidptosis, this ER stress signaling is identified as a secondary response to upstream disulfide accumulation rather than a primary causative driver. As unreduced disulfides build up, they physically disrupt the ER’s protein-folding machinery, thereby triggering the P-eIF2α → ATF4 → ATF3 axis as a late-stage stress signal.

Interestingly, this secondary ER stress response may serve a compensatory, protective role, as its inhibition has been shown to sensitize cells to disulfidptosis [35]. At the structural level, the Wiskott-Aldrich syndrome protein-family verprolin-homologous protein (WAVE) regulatory complex (WRC) and the small GTPase Rac1 act as positive regulators of disulfidptosis [8,44,46]. Rac-WRC-mediated lamellipodia formation generates a branched actin network that appears uniquely vulnerable to the disulfide crosslinking and physical collapse that defines this RCD program [8,9].

## 3. The SLC7A11 Paradox—Metabolic Dependency and Metal-Modulated Triggers

While the preceding sections outlined the conceptual divergence between regulated death modes, the following analysis establishes the definitive biochemical execution of disulfidptosis, centralizing the interplay between SLC7A11-mediated transport and reductive exhaustion. The dual nature of SLC7A11 as both a survival factor and a lethal vulnerability constitutes a fundamental paradox in cancer metabolism. As introduced in the preceding sections, the detailed biochemical execution of this transition is established here through the interplay of nutrient availability and reductive demand. Functioning as a cystine/glutamate antiporter, SLC7A11 imports extracellular cystine into the cell while exporting glutamate [8,47,48]. Under physiological conditions, this imported cystine is rapidly reduced to cysteine, the rate-limiting precursor for GSH synthesis [29,47]. This SLC7A11-GSH-GPX4 axis serves as the primary defense against ferroptosis, as GPX4 utilizes GSH to neutralize lethal lipid peroxides [26,29,30,47]. However, this anti-ferroptotic shield imposes a profound metabolic hyper-dependency: the reduction of cystine to cysteine is a high-demand, NADPH-consuming reaction [22,26]. In malignant cells, the cytosolic NADPH pool is primarily replenished through the glucose-dependent pentose phosphate pathway (PPP) [8,49,50,51,52]. Consequently, under conditions of glucose starvation or pharmacological GLUT inhibition, the NADPH supply is severed, inducing an acute deficit of this reducing equivalent [8,22]. SLC7A11-high cells continue to import cystine but lack the reductive capacity to process it, leading to the systemic accumulation of intracellular disulfides such as cystine and glutamyl-cystine [8,22,23,53]. This acute disulfide stress triggers the formation of aberrant intermolecular disulfide bonds in actin cytoskeleton proteins, resulting in systemic cytoskeletal collapse and the induction of disulfidptosis [8,53,54].

**Figure 3:**
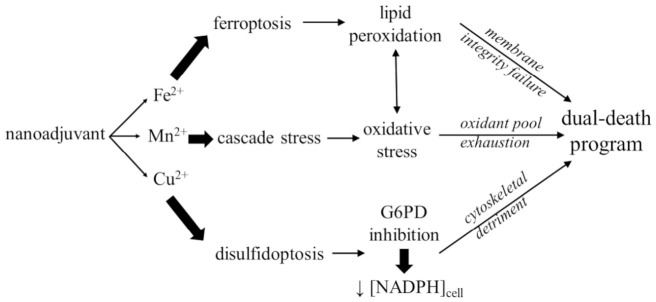
Metal-driven orchestration of a synergistic dual-death program targeting the SLC7A11 axis. The schematic illustrates the therapeutic exploitation of the SLC7A11 metabolic vulnerability using a multifunctional metal-based nanoadjuvant. Upon endocytosis and intracellular release, the metallic components (Fe^2+^, Mn^2+^, and Cu^2+^) trigger a three-pronged biochemical assault. (**Top**) The ferroptosis axis: Fe^2+^ catalyzes the Fenton reaction, driving lipid peroxidation (LPO) of the PUFA bilayer, ultimately resulting in membrane integrity failure. (**Center**) The cascade stress hub: Mn^2+^ amplifies the lethal environment by generating sustained oxidative stress, which feeds into the LPO pathway and leads to systemic antioxidant pool exhaustion. (**Bottom**) The disulfidptosis axis: Cu^2+^ overload acts as a metabolic “off-switch” by inhibiting G6PD, the rate-limiting enzyme of the PPP. The resulting precipitous drop in intracellular NADPH levels prevents the reduction of imported cystine, inducing acute disulfide stress and catastrophic cytoskeletal detriment. The convergence of these mechanisms ensures the irreversible execution of an integrated dual-death program.

While ferroptosis is classically defined as an iron-dependent process, emerging evidence increasingly links disulfidptosis to a broader landscape of metal dyshomeostasis (Figure 3). In ferroptosis, iron is the primary catalyst; intracellular labile Fe^2+^ pools catalyze the Fenton reaction with H_2_O_2_ to generate highly toxic hydroxyl radicals [55,56], which initiate the membrane-destroying lipid peroxidation [28,57]. However, disulfidptosis appears susceptible to modulation by other transition metals, notably copper and manganese. Recent studies suggest that copper ions can inhibit glucose-6-phosphate dehydrogenase (G6PD), the rate-limiting enzyme of the PPP and a primary source of cellular NADPH, thereby depleting the reductive pool and triggering disulfidptosis [32,58]. While the inhibition of G6PD by copper ions has been documented [59,60], the precise coordination chemistry governing this interaction remains an active area of investigation. It is hypothesized that Cu^2+^ may interfere with the enzyme’s active site or induce structural modifications that impair the PPP. However, a critical question remains: is this inhibition quantitatively sufficient to act as the primary trigger for disulfidptosis? In clinical contexts, it is more likely that copper-induced PPP disruption acts as a synergistic “metabolic amplifier” rather than a solitary driver, working in tandem with the acute NADPH demands imposed by high-velocity SLC7A11 transport.

This has led to the proposal of dual-death models, where copper overload simultaneously facilitates cuproptosis and disulfidptosis by disrupting mitochondrial tricarboxylic acid (TCA) cycle proteins and metabolic reduction systems [32,61]. Similarly, Mn^2+^-based nanoadjuvants have been shown to induce disulfidptosis by amplifying oxidative stress and depleting NADPH through cascade reactions [36]. Although disulfidptosis is not primarily driven by iron, SLC7A11 expression levels can influence the labile iron pool [16]. Furthermore, tumor suppressors like BAP1 (BRCA1 (breast cancer gene 1)-associated protein 1), an enzyme that restricts SLC7A11 production, may modulate disulfidptosis sensitivity through pathways that overlap with traditional iron metabolism [62,63,64].

**Figure 4:**
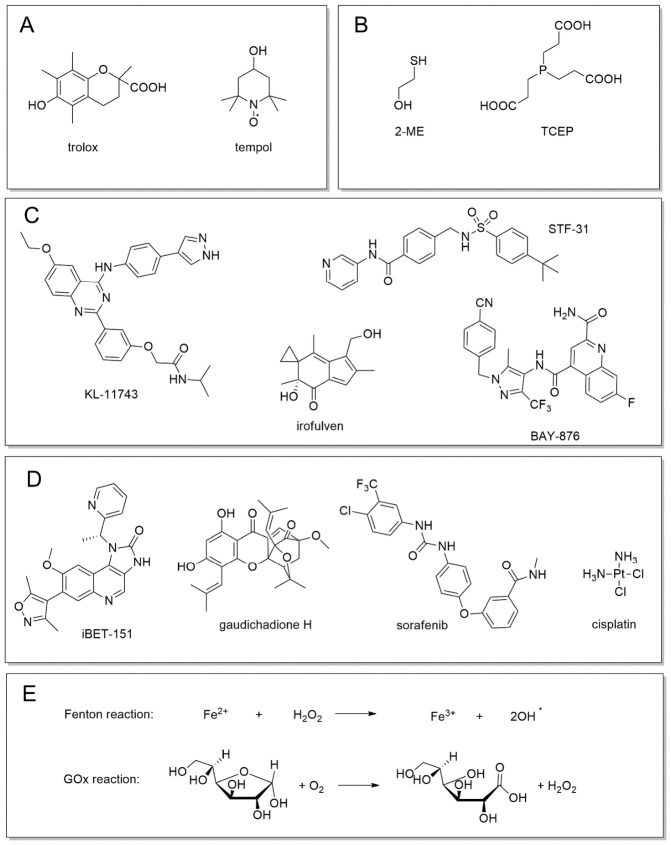
Chemical modulators of the SLC7A11 metabolic axis. (**A**) Ferroptosis inhibitors: traditional RTAs that neutralize lipid peroxyl radicals. (**B**) Disulfidptosis rescuers: thiol-based (2-mercaptoethanol; 2-ME) and phosphine-based (tris 2-carboxyethyl phosphine; TCEP) reducing agents that maintain protein cysteinyl residues in a reduced state, preventing F-actin cross-linking. (**C**) Metabolic inducers: selective inhibitors of GLUTs that trigger NADPH exhaustion in SLC7A11-high cells. (**D**) Synergistic modulators: clinical chemotherapeutics and targeted inhibitors (e.g., nuclear factor erythroid 2-related factor 2 (NRF2)-modulators and bromodomain and extra-terminal motif (BET)-inhibitors) that sensitize the SLC7A11 axis. (**E**) Executioners of collapse: primary biochemical cascades, including the iron-catalyzed Fenton reaction and the glucose oxidase (GOx)-mediated depletion of glucose, which drive the transition from oxidative stress to catastrophic structural collapse.

The divergent nature of these death modes is further clarified by their unique biochemical signatures and the specific coordination chemistry of their pharmacological rescuers (Figure 4). These interactions are governed by distinct redox kinetics that differentiate oxidative protection from reductive restoration. In ferroptosis, radical-trapping antioxidants (RTAs) function as chain-breaking agents to slow the rate of lipid autoxidation (Figure 4A). These molecules kinetically outcompete polyunsaturated fatty acids for peroxyl radicals, thereby halting the self-propagating oxidative chain reaction before membrane integrity is compromised [8,29,30,65].

In sharp contrast, disulfidptosis rescuers operate through direct thiol-disulfide exchange or phosphine-mediated reduction (Figure 4B). Reducing agents such as 2-ME or TCEP do not act on radical species; instead, they provide a pool of exogenous electrons to kinetically outpace the formation of aberrant protein cross-links. By maintaining structural proteins like FLNA and MYH9 in a reduced thiol state, these agents prevent the physical disulfide bonding that drives cytoskeletal collapse (Figure 4B). This distinction confirms that while ferroptosis is an oxidative failure of the membrane, disulfidptosis is a kinetic failure of the cell’s reductive machinery to manage its systemic disulfide burden. Consequently, while ferroptosis is defined by the accumulation of lipid ROS such as malondialdehyde (MDA), disulfidptosis is ROS-independent and characterized by a precipitous rise in the NADP+/NADPH ratio [8,9,35,66].

Beyond simple rescue, the pharmacological landscape includes potent inducers and synergistic modulators that exploit the SLC7A11 metabolic vulnerability (Figure 4C–E). For instance, metabolic induction is achieved through selective GLUT inhibitors, which starve SLC7A11-high cells of glucose, effectively severing the NADPH supply from the pentose phosphate pathway (Figure 4C). This metabolic blockade can be further amplified by the administration of GOx, which depletes intracellular glucose to trigger a catastrophic transition from manageable oxidative stress to structural collapse (Figure 4E).

Furthermore, the therapeutic window for disulfidptosis is expanded by transcriptional modulators that sensitize the SLC7A11 axis (Figure 4D). Targeted inhibitors such as NRF2-modulators function as potent sensitizers by disrupting the antioxidant response element transcriptional programs, thereby lowering the metabolic threshold at which cells can tolerate cystine-induced stress. Similarly, BET-inhibitors (e.g., iBET-151) interfere with the epigenetic regulation of SLC7A11, demonstrating that the induction of disulfidptosis is not merely a consequence of nutrient deprivation but a targetable program regulated at the level of gene expression.

Morphologically, while ferroptosis manifests as shrunken mitochondria with increased membrane density [39,40,41], the definitive marker for disulfidptosis is actin cytoskeleton collapse [8,53,54]. This is evidenced by the formation of intermolecular disulfide bonds, detectable via non-reducing Western blot, in structural proteins such as FLNA, filamin B (FLNB), and MYH9, which cause the F-actin network to contract and detach from the plasma membrane [8,9,42,43].

This intersection of pathways is mirrored in a shared regulatory landscape of crossover genes identified through multi-omics analyses, such as the ferroptosis- and disulfidptosis-related gene (FDRG) and disulfidptosis- and ferroptosis-related gene prognostic score (DRGPS) models (Table 3). SLC7A11 remains the primary regulator, acting as a switch that suppresses ferroptosis while promoting disulfidptosis [67]. Its chaperone protein, SLC3A2 (solute carrier family 3 member 2, also known as CD98hc), has similarly emerged as a critical prognostic marker across lung adenocarcinoma (LUAD), colorectal (CRC), and hepatocellular (HCC) carcinomas [11,47,68,69,70,71]. Other key shared regulators include leucine-rich pentatricopeptide repeat-containing protein (LRPPRC), a mitochondrial gene linked to risk signatures in CRC and HCC [72,73], and the mitochondrial complex I subunits NDUFA11 (NADH:ubiquinone oxidoreductase subunit A11) and NDUFS1 (NADH:ubiquinone oxidoreductase core subunit S1), which correlate significantly with survival in both contexts [11,71]. Additionally, glutathione-disulfide reductase (GSR) directly links the reduction of glutathione disulfide (GSSG), which protects against ferroptosis, to the overall cellular disulfide burden, which regulates disulfidptosis [26,74]. Notable tumor suppressors like the charged multivesicular body protein 6 (CHMP6) in CRC [75] and the actin-remodeling protein inverted formin 2 (INF2), particularly in ovarian cancer [76], further bridge these programs. Finally, while GPX4 is the master regulator of ferroptosis, its frequent modulation during disulfidptosis induction suggests a broader, more catastrophic collapse of the cell’s integrated antioxidant systems [29,77].

## 4. Clinical and Therapeutic Implications

Multi-omic analyses and therapeutic modeling across current literature indicate that disulfidptosis and ferroptosis represent distinct yet therapeutically synergistic metabolic vulnerabilities, particularly in SLC7A11-overexpressing tumors [11,22,79]. The clinical significance of these cell death modalities is underscored by their evolving roles in prognostic stratification, the remodeling of the immune microenvironment, and the pharmacological reversal of multi-drug resistance [11,71,76] (Table 4). The practical application of these pathways is perhaps most evident in the development of sophisticated multi-gene risk scores tailored to specific malignancies. In HCC, the DRGPS—comprising the transporter SLC7A11, the matrix protein MATN3 (matrilin 3), the glycoprotein CLEC3B (tetranectin), the cell-cycle regulator CCNJL (cyclin J-like), and the antioxidant enzyme PON1 (paraoxonase 1)—serves as a robust predictor, where high scores correlate with advanced TNM (tumor, node, metastasis) stages and significantly reduced overall survival [71,80].

Similarly, the DFRG signature in LUAD utilizes the metabolic enzyme GMPR (guanosine monophosphate reductase), the transport protein MCFD2 (multiple coagulation factor deficiency 2), the mitochondrial ribosomal protein MRPL13, and the transcription factor SALL2 (spalt-like transcription factor 2) as optimal predictors [11]. In CRC, an eight-gene FDRG score effectively stratifies patients into high-risk groups characterized by elevated recurrence rates [75]. This model reflects the biological diversity of CRC pathogenesis by integrating pro-oxidant engines such as NOX4 (NADPH oxidase 4), ALOX12 (arachidonate 12-lipoxygenase), and NOS2 (nitric oxide synthase 2) with regulators of membrane trafficking and immune signaling. Specifically, the signature distinguishes these metabolic factors from the membrane-trafficking protein CHMP6 (charged multivesicular body protein 6)—a component of the ESCRT-III complex involved in endosome-to-lysosome transport—and the immune-modulatory factors PTPN6 (protein tyrosine phosphatase non-receptor type 6) and MMD (monocyte to macrophage differentiation-associated protein). These are further coordinated with the ER-stress mediator DDIT3 (DNA damage inducible transcript 3) and the metabolic factor COX4I2 (cytochrome c oxidase subunit 4I2).

Furthermore, a pan-cancer DRG (disulfidptosis-related gene) model utilizing *ACTN4* (α-actinin-4), *ACTB* (actin β), *FLNA*, *FLNB*, and *INF2* has revealed that elevated scores in ovarian and uterine cancers are strong indicators of poor prognosis [76,81]. Throughout these models, *SLC7A11* and *INF2* consistently emerge as independent risk factors; high SLC7A11 expression not only promotes proliferation and migration but also creates a terminal dependency on glucose [71,76]. Conversely, genes such as *GMPR* and *CHMP6* function as tumor suppressors, with their downregulation correlating with increased malignancy [75] (Table 4).

**Figure 5:**
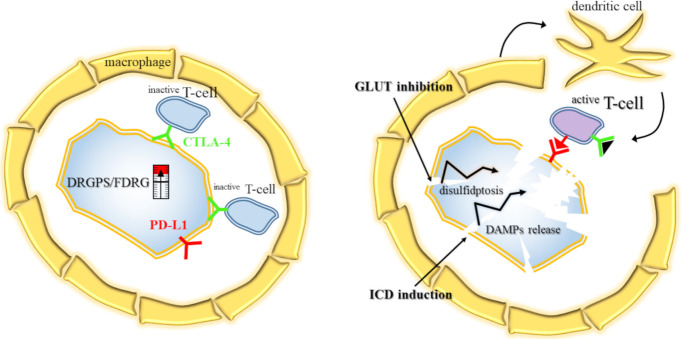
Clinical transformation of the SLC7A11-high TME. (**Left**) The high-risk baseline: patients with elevated DRGPS/FDRG scores exhibit an immunologically “cold” or excluded phenotype. The SLC7A11-overexpressing tumor cell is shielded by upregulated immune checkpoints (PD-L1, CTLA-4) and a physical barrier of M2 macrophages. Infiltrating CD8+ T-cells remain in an exhausted state, unable to penetrate the metabolic and molecular defenses. (**Right**) The primed response: therapeutic intervention via GLUT inhibition (triggering disulfidptosis) and ICD induction initiates a “loud” metabolic collapse. The resulting release of damage-associated molecular patterns (DAMPs) facilitates dendritic cell (DC) maturation and recruitment. These mature DCs orchestrate the infiltration of effector CD8+ T-cells, which fragment the macrophage wall and achieve total tumor ablation, effectively converting the TME into an immune-hot niche sensitized for immunotherapy.

Beyond survival metrics, the underlying metabolic state of SLC7A11-high cells profoundly reshapes the tumor microenvironment (TME) and dictates immune infiltration patterns (Figure 5). High-risk profiles, as defined by FDRG or DRGPS scores, generally manifest an immunosuppressive TME characterized by the recruitment of M2 macrophages, myeloid-derived suppressor cells, and regulatory T cells [71,75]. These high-risk groups frequently exhibit upregulation of immune checkpoints, including PD-L1 (programmed death-ligand 1), PD-1 (programmed cell death protein 1), and CTLA-4 (cytotoxic T-lymphocyte-associated protein 4); yet, they often present higher TIDE (tumor immune dysfunction and exclusion) scores, suggesting a diminished likelihood of clinical response to monotherapeutic checkpoint inhibition [71,75,76,82].

The induction of disulfidptosis via specialized nanoadjuvants, such as BMP-Au (biomineralized manganese oxide-phospholipid-gold), offers a strategic pathway to prime the immune system. By triggering immunogenic cell death (ICD), these interventions promote the release of damage-associated molecular patterns, including HMGB1 (high mobility group box 1) and ATP, alongside the surface exposure of calreticulin [22,36]. This process activates the cGAS-STING signaling axis—named for the cyclic guanosine monophosphate-adenosine monophosphate synthase enzyme and the stimulator of interferon genes protein. This pathway senses cytoplasmic DNA to facilitate dendritic cell maturation and increase the infiltration of CD8+ cytotoxic T cells (Figure 5). This transformation effectively converts immune-excluded or non-inflamed microenvironments into T-cell-inflamed niches, thereby enhancing the efficacy of immunotherapy [22,36].

The clinical imperative to harness these vulnerabilities is further underscored by the potential to bypass traditional chemoresistance. Clinically relevant agents such as Sorafenib for HCC, Cisplatin for bladder cancer, and Irofulven for ovarian cancer (OV) have been identified as potential modulators of these pathways [47,71,76]. Because the high-cystine environment of SLC7A11-high cells creates an intrinsic requirement for glucose-derived NADPH, selective GLUT inhibitors—including BAY-876, KL-11743, and STF-31—can be deployed to trigger catastrophic disulfidptosis [9,29,35,53]. This strategy is particularly effective in reversing drug resistance; for instance, in cisplatin-resistant bladder cancer—where SLC7A11 is typically upregulated as a protective mechanism—an orchestrated glucose depletion and redox-disruption strategy (e.g., utilizing M@Cys-Gox-Mn nanoparticles) can achieve complete tumor regression [47]. Furthermore, novel modulators like Gaudichaudione H (GH) and iBET-151 have demonstrated the ability to sensitize cells to disulfidptosis by modulating the NRF2-SLC7A11 axis or super-enhancer activity, respectively [53,78].

Importantly, as established by the central theme of metal dyshomeostasis, innovative theranostic platforms are now utilizing metals as both therapeutic triggers and real-time monitoring agents. Iron-mediated synergy is achieved through platforms like Fe-Bi-succinoyl-porphyrin metal–organic frameworks (MOFs) and FeOOH@Fe-Ap@Au nanoshuttles, which deliver iron to catalyze the Fenton reaction while concurrently utilizing GOx to deplete glucose and trigger disulfidptosis [22,29]. This approach also facilitates radiosensitization through high-Z elements like Bi, which increases radiation energy deposition [22]. Additionally, copper-driven dual-death models utilize copper-selenium-tripeptide nanoparticles to specifically inhibit G6PD, thereby depleting NADPH and binding to lipoylated TCA cycle proteins to trigger simultaneous cuproptosis and disulfidptosis [30,32]. Manganese-based adjuvants (e.g., BMP-Au) further amplify this effect by catalyzing Fenton-like reactions and serving as cofactors to enhance cGAS-STING activation following disulfidptosis-induced DNA damage [36]. Finally, iron-based hybrid nanoenzymes (M@GOx/Fe-HMON) enable a comprehensive theranostic approach, allowing for MRI-guided therapy and the real-time monitoring of metabolic responses and tumor accumulation [31].

## 5. Future Directions and Research Gaps

**Figure 6:**
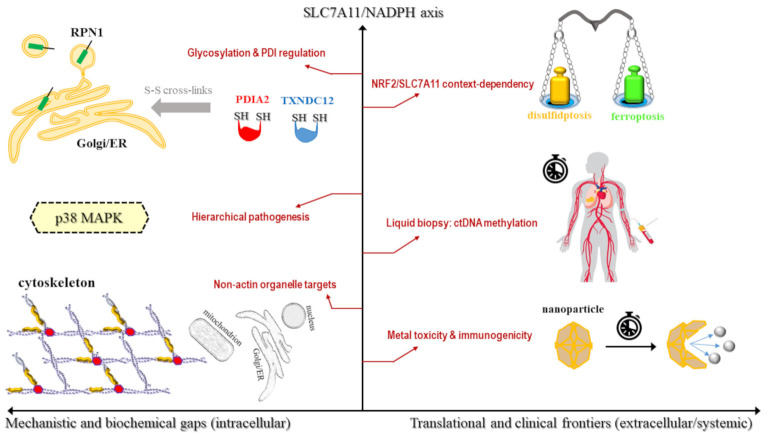
Uncharted terrains: the mechanistic and clinical frontiers of disulfidptosis. The schematic outlines the critical research gaps and future directions within the SLC7A11-disulfidptosis regulatory framework. (**Left**) Intracellular mechanistic gaps: questions remain regarding whether N-glycosylation or PDI-mediated (e.g., PDIA2, TXNDC12) protein folding acts as the “master switch” for initiating catastrophic disulfide stress (S-S cross-linking). The role of p38 MAPK as a primary driver versus secondary bystander remains to be elucidated, as does the potential for lethal disulfide targets beyond actin, such as the mitochondria, nucleus, or ER/Golgi network (where RPN1 may act as a chaperone). (**Right**) Translational frontiers: The NRF2/SLC7A11 context-dependency highlights the context-dependent nature of SLC7A11, where its induction confers ferroptosis protection while simultaneously increasing disulfidptosis sensitivity (balanced scales). Clinical implementation requires the development of liquid biopsy–ctDNA methylation platforms for the dynamic monitoring of SLC7A11 methylation. Additionally, long-term metal retention and chronic immunotoxicity following nanoparticle-mediated delivery remain primary toxicological concerns.

The discovery of disulfidptosis provides a significant breakthrough in understanding SLC7A11-mediated cell death; however, its full characterization is hindered by substantial mechanistic and clinical gaps (Figure 6). Although the role of the SLC7A11-NADPH-actin cytoskeleton axis is well-established, several mechanistic lacunae remain in the biochemical implementation of the death program. For instance, while it is hypothesized that disulfidptosis involves disulfide bonding in multiple proteins beyond the actin network, these targets have not yet been definitively proven as primary mediators [8]. Furthermore, although functional genomics have identified ribophorin I (RPN1)—a structural protein of the ER involved in protein folding and maturation—as a potent suppressor of disulfidptosis, the underlying mechanism remains unclear. Its protective role appears independent of its traditional functions in protein modification, suggesting it may act as a specialized chaperone that prevents the premature cross-linking of SLC7A11 or other sensitive cysteine-rich proteins [53].

Importantly, the regulatory roles of protein disulfide isomerases (PDIs) such as PDIA2 and TXNDC12 in governing the pathogenic crosslinking of cytoskeletal proteins remain largely theoretical [83]. While these enzymes are biologically plausible candidates for facilitating intermolecular disulfide exchange under conditions of reductive exhaustion, direct experimental evidence validating their necessity as executioners of disulfidptosis is currently lacking.

This knowledge gap extends to signaling circuits; it remains to be determined which specific pathways act as primary drivers versus secondary responses to the catastrophic disulfide collapse (Table 5). Ultimately, the precise molecular crosstalk between disulfidptosis and other regulated death programs, such as autophagy or the integrated ER stress response, remains elusive [10].

Beyond these proximal biochemical steps, the framework connecting disulfidptosis to metal dyshomeostasis requires more rigorous validation. While current models suggest that copper ions can trigger disulfidptosis by inhibiting G6PD and depleting the NADPH pool, it remains unclear whether copper-induced disruption of the PPP is potent enough to serve as the primary driver of death in a clinical setting [84]. Authors in the field explicitly acknowledge that further exploring the mechanisms underlying copper overload-induced death remains an essential yet challenging endeavor. Similarly, the role of iron in disulfidptosis is largely characterized as indirect, and the therapeutic use of manganese-based nanoadjuvants is in its infancy; crucially, whether these metal-based platforms can sustain long-term immune memory in human patients is currently unknown [36].

This biochemical complexity is further mirrored in the significant research variations and contradictions observed across different cancer types and multi-omic models (Table 5). For example, a prognostic signature comprising *GMPR*, *MCFD2*, *MRPL13*, and *SALL2* has shown high predictive value in LUAD, but its relevance to other malignancies like HCC or acute myeloid leukemia (AML) has not been established [85]. Large-scale pan-cancer analyses admit that substantial cohort heterogeneity, stemming from differences in tumor purity and sample sizes, can skew associations and reduce the generalizability of current disulfidptosis risk scores [76]. Furthermore, a notable paradox exists regarding NRF2; while it is a classical suppressor of ferroptosis, emerging evidence suggests that natural compounds like GH can utilize the NRF2-SLC7A11 axis to actually sensitize cells to disulfidptosis, indicating that NRF2 may play diametrically opposite roles depending on the therapeutic context [86] (Figure 6).

These research variations directly translate into hurdles for clinical implementation, where the transition from laboratory discovery to patient care faces significant practical obstacles. A primary concern involves the prolonged in vivo retention of metal-based nanomaterials, which may lead to unforeseen immunotoxicity or chronic inflammatory responses in major organs [87,88]. For small-molecule sensitizers like GH, the fundamental pharmacokinetics, biodistribution, and metabolic processes remain poorly understood, complicating the assessment of their therapeutic viability [78]. Additionally, current datasets like The Cancer Genome Atlas (TCGA) lack the temporal sampling required for dynamic biomarker tracking. This suggests a critical need for the development of liquid biopsy techniques, such as monitoring SLC7A11 methylation in circulating tumor DNA (ctDNA), to follow disulfidptosis activity in real-time (Figure 6).

In light of these challenges, five pressing questions must be prioritized to move the field forward. First, researchers must determine the specific thresholds of disulfide-bond formation in actin cytoskeleton proteins required to definitively trigger the irreversible phase of disulfidptosis [9]. Second, it remains to be seen if NADPH depletion is a selective trigger for actin crosslinking or if other sulfhydryl-containing proteins in different organelles undergo similar pathogenic modifications. Third, the definitive role of mitochondria in disulfidptosis must be clarified to distinguish it from its essential roles in ferroptosis and apoptosis. Fourth, strategies must be developed to overcome the SLC7A11-dependency of disulfidptosis, enabling dual-target therapies that are effective even in SLC7A11-low or chemoresistant tumors [47]. Finally, future clinical trials must determine whether susceptibility to disulfidptosis can serve as a valid biomarker to select patients for treatment with GLUT inhibitors or other metabolic therapies [9]. By addressing these gaps, the field can transition from characterizing a biological paradox to engineering a new class of metal-driven precision medicines.

## 6. Conclusions: Metal Dyshomeostasis as the Final Frontier of RCD

The comprehensive synthesis of the current evidence reveals that the intersection of ferroptosis and disulfidptosis is fundamentally governed by metal dyshomeostasis, which serves as the primary driver for executing SLC7A11-mediated cell death. This transition from oxidative membrane damage to structural and metabolic collapse represents a final frontier in precision oncology, offering a robust strategy for bypassing traditional chemoresistance. This pivot redefines the SLC7A11 axis from a simple antioxidant shield into a targetable metabolic liability, marking a paradigm shift in the strategic exploitation of regulated cell death.

This synergy is further exemplified by the emergence of multifunctional therapeutic platforms, such as MOFs and hybrid nanoenzymes, which utilize metal ions to synchronously evoke integrated death programs. These platforms exploit the SLC7A11 axis by inducing a systemic redox collapse that successfully bypasses conventional apoptosis resistance in refractory malignancies. Moreover, the integration of metal-based nanoadjuvants demonstrates that metal-coordinated strategies act as both cytotoxic triggers and potent immunomodulatory cofactors. By enhancing the remodeling of the immunosuppressive TME, these strategies promote long-term anti-tumor immunity, transforming the metabolic landscape of the tumor into a site of robust immune engagement.

Ultimately, the strategic modulation of metal ion homeostasis provides a robust framework to exploit the SLC7A11 metabolic paradox. By integrating targeted metabolic disruption with metal-catalyzed attacks, we move beyond the biochemical mechanics of cell death and toward a unified therapeutic framework. This framework enables the eradication of heterogeneous and drug-resistant tumor niches, suggesting that the future of cancer therapy lies in our ability to systematically turn a cell’s primary defense mechanism into its defining vulnerability.

## Figures and Tables

**Table 1:** Biochemical and morphological divergence of SLC7A11-mediated cell death programs.

Feature	Ferroptosis	Disulfidptosis
**Primary driver**	iron-catalyzed lipid peroxidation	disulfide stress/NADPH exhaustion
**Master regulator**	GPX4/SLC7A11	SLC7A11/GLUTs
**Metabolic state**	iron overload/GSH depletion	glucose starvation/NADPH deficit
**Morphological marker**	shrunken mitochondria	actin cytoskeleton collapse
**Effect of reactive oxygen species (ROS) scavengers**	inhibits (e.g., liproxstatin-1)	no effect (ROS-independent)
**Effect of reducing agents**	limited	inhibits (e.g., 2-ME, TCEP)
**Effect of GLUT inhibitors**	often suppresses (lower metabolism)	triggers/accelerates

**Table 2:** Mechanistic and morphological distinctions in the execution of ferroptosis and disulfidptosis. Comparison of the biochemical “points of no return”, structural targets, and regulatory signaling axes. Note the “cystine paradox”, where cystine serves as a requisite for disulfidptosis execution while acting as a survival factor against ferroptosis [8,9,29,35]. ACSL4: acyl-CoA synthetase long-chain family member 4; LPCAT3: lysophosphatidylcholine acyltransferase 3; PUFA-PL: polyunsaturated fatty acid-containing phospholipid.

Category	Ferroptosis	Disulfidptosis
**Primary structural target**	PUFAs in membranes [29,30]	Actin cytoskeleton proteins (e.g., FLNA, MYH9) [8,9]
**Metabolic “point of no return”**	Lipid hydroperoxide accumulation [31,32]	Acute NADPH deficit and disulfide stress [8,36]
**Role of ER**	Site of PUFA-phospholipid synthesis (ACSL4/LPCAT3) [29]	Activation of P-eIF2*α*/ATF4/ATF3 stress axis [35]
**Cytoskeletal regulators**	Minimal known direct regulation	Positive regulation by Rac1 and WRC [8,9]
**Cystine sensitivity**	Cystine starvation triggers death [8]	Cystine starvation suppresses death [8,9]
**ATP requirement**	Often ATP-independent	Independent of ATP depletion [8,9]

**Table 3:** Clinical and molecular integration of the SLC7A11-regulated crossover genes.

Genetic Marker/Score	Functional Role in SLC7A11 Axis	Clinical Implications & Cancer Types	Key Refs
**DRGPS/FDRG scores**	Composite multi-omic RCD signatures	Prognostic for overall survival and Immunotherapy response	[11,71]
***SLC3A2*** **(CD98hc)**	SLC7A11 chaperone and stabilizer	Prognostic marker in LUAD, CRC, and HCC	[8,11,47]
***GSR***	Mediates GSSG reduction to GSH	Integrated defense against both death modes	[26,78]
***LRPPRC***	Mitochondrial RNA stability regulator	Associated with risk signatures in CRC and HCC	[11,75]
***INF2***	Actin-remodeling/nucleation protein	Mediator of cytoskeletal collapse in ovarian cancer	[76]
***NDUFA11/NDUFS1***	Mitochondrial complex I subunits	Survival correlation in multiple solid tumors	[11,71]

**Table 4:** Validated prognostic signatures and genomic correlations across malignancies. Summary of multi-gene risk scores (DRGPS, DFRG, and FDRG) developed to stratify patient outcomes. These models integrate multidimensional factors, including metabolic dependencies, membrane trafficking, and immune-modulatory signaling, with genomic mutations, including tumor protein p53 (*TP53*) and titin (*TTN*), to identify high-risk cohorts characterized by poor overall survival and therapeutic resistance [11,71,75,76].

Signature	Cancer Type	Constituent Genes (Key Predictors)	Clinical/Genomic Correlation
**DRGPS**	HCC	*SLC7A11*, *MATN3*, *CLEC3B*, *CCNJL*, *PON1*	Advanced TNM stage; reduced OS
**DFRG**	LUAD	*GMPR*, *MCFD2*, *MRPL13*, *SALL2*	*TP53* and *TTN* genomic mutations
**FDRG**	CRC	Metabolic: *NOX4*, *ALOX12*, *NOS2*, *COX4I2*; Trafficking: *CHMP6*; Immune/Stress: *PTPN6*, *MMD*, *DDIT3*	Elevated recurrence rates; reflects the convergence of metabolic failure, membrane trafficking defects, and immune signaling.
**Pan-cancer DRG**	OV/UCEC	*ACTN4*, *ACTB*, *FLNA*, *FLNB*, *INF2*	Poor prognosis; *INF2* as independent risk factor

**Table 5:** Identified research gaps and clinical obstacles.

Category	Key Research Gap	Future Requirement
**Molecular machinery**	Validating non-actin substrates and the hypothetical role of PDIs/RPN1	Use of high-resolution mass spectrometry to map the disulfidome and confirm enzymatic necessity via CRISPR-Cas9 screens.
**Signaling circuits**	Distinguishing primary signaling drivers versus secondary stress responses	Implementation of time-resolved phosphoproteomics to establish the kinetic hierarchy of p38 MAPK and ER-stress crosstalk.
**Pharmacological**	Under-characterized pharmacokinetics, biodistribution, and metabolism of GH and iBET-151	Detailed PK/PD modeling and biodistribution studies to assess therapeutic viability and systemic safety profiles.
**Diagnostic**	Static nature of TCGA datasets lacks temporal and dynamic depth	Transition to liquid biopsy (ctDNA) platforms to track real-time epigenetic shifts, such as SLC7A11 methylation.
**Toxicological**	Long-term systemic retention and clearance of metal-based nanomaterials	Longitudinal in vivo toxicity assays focusing on organ-specific accumulation and chronic immunotoxicity markers.

## Data Availability

No new data were created or analyzed in this study.

## Abbreviations

The following abbreviations are used in this manuscript:

2-ME	β-mercaptoethanol
4-HNE	4-hydroxynonenal
ACSL4	Acyl-CoA synthetase long-chain family member 4
ACTB	Actin β
ACTN4	α-actinin-4
ALOX12	Arachidonate 12-lipoxygenase
AML	Acute myeloid leukemia
ATF3/4	Activating transcription factor 3/4
ATP	Adenosine triphosphate
BAP1	BRCA1-associated protein 1
BET	Bromodomain and extra-terminal motif
BMP	Biomineralized manganese oxide-phospholipid
BRCA1	Breast cancer gene 1
CCNJL	Cyclin J-like
cGAS-STING	Cyclic GMP-AMP synthase-stimulator of interferon genes
CHMP6	Charged multivesicular body protein 6
CLEC3B	C-type lectin domain family 3 member B (tetranectin)
COX4I2	Cytochrome c oxidase subunit 4I2
CRC	Colorectal cancer
ctDNA	Circulating tumor DNA
CTLA-4	Cytotoxic T-lymphocyte-associated protein 4
DAMPs	Damage-associated molecular patterns
DC	Dendritic cell
DDIT3	DNA damage inducible transcript 3
DFRG	Disulfidptosis- and ferroptosis-related gene
DRGPS	Disulfidptosis- and ferroptosis-related gene prognostic score
ER	Endoplasmic reticulum
FDRG	Ferroptosis- and disulfidptosis-related gene
FLNA/B	Filamin A/B
G6PD	Glucose-6-phosphate dehydrogenase
GH	Gaudichaudione H
GLUT	Glucose transporter
GMPR	Guanosine monophosphate reductase
GOx	Glucose oxidase
GPX4	Glutathione peroxidase 4
GSH	Glutathione (reduced)
GSR	Glutathione-disulfide reductase
GSSG	Glutathione disulfide (oxidized)
HCC	Hepatocellular carcinoma
HMGB1	High mobility group box 1
ICD	Immunogenic cell death
INF2	Inverted formin 2
LPCAT3	Lysophosphatidylcholine acyltransferase 3
LPO	Lipid peroxidation
LRPPRC	Leucine-rich pentatricopeptide repeat-containing protein
LUAD	Lung adenocarcinoma
MATN3	Matrilin 3
MCFD2	Multiple coagulation factor deficiency 2
MDA	Malondialdehyde
MMD	Monocyte to macrophage differentiation-associated protein
MOF	Metal–organic framework
MRPL13	Mitochondrial ribosomal protein L13
MYH9	Myosin heavy chain 9 (non-muscle myosin II)
NADPH	Nicotinamide adenine dinucleotide phosphate
NDUFA11/S1	NADH:ubiquinone oxidoreductase subunit A11/S1
NOS2	Nitric oxide synthase 2
NOX4	NADPH oxidase 4
NRF2	Nuclear factor erythroid 2-related factor 2
OV	Ovarian cancer
P-eIF2α	Phosphorylated eukaryotic initiation factor 2 alpha
PDI	Protein disulfide isomerase
PDIA2	Protein disulfide isomerase family A member 2
PD-1/L1	Programmed cell death protein 1/ligand 1
PON1	Paraoxonase 1
PPP	Pentose phosphate pathway
PTPN6	Protein tyrosine phosphatase non-receptor type 6
PUFA	Polyunsaturated fatty acid
PUFA-PL	Polyunsaturated fatty acid-containing phospholipid
RCD	Regulated cell death
ROS	Reactive oxygen species
RPN1	Ribophorin I
RTA	Radical-trapping antioxidant
SALL2	Spalt-like transcription factor 2
SLC3A2	Solute carrier family 3 member 2, also known as CD98hc
SLC7A11 (xCT)	Solute carrier family 7 member 11
$x_{c}^{-}$	Heterodimeric amino acid transporter (SLC7A11 + SLC3A2)
TCA	Tricarboxylic acid
TCGA	The Cancer Genome Atlas
TCEP	Tris(2-carboxyethyl)phosphine
TIDE	Tumor immune dysfunction and exclusion
TME	Tumor microenvironment
TNM	Tumor, Node, Metastasis (staging system)
TP53	Tumor protein p53
TTN	Titin
TXNDC12	Thioredoxin domain-containing 12
UCEC	Uterine corpus endometrial carcinoma
WAVE	Wiskott-Aldrich syndrome protein-family verprolin-homologous protein
WRC	WAVE regulatory complex
